# National Health

**Published:** 1861-04

**Authors:** 


**NATIONAL HEALTH** would be promoted and the pulse of the country would fall rapidly towards its normal action by taking five hundred of the leading political demagogues, and an equal quantity of reckless, unprincipled, and time-serving editors; then put one of each in a sack capable of holding four, sling them across mules, trot them off to Cincinnati, fill their pockets with corn, empty them out, tied hand and foot into a pork-lot, and let the pigs root them into the Ohio river, and there let them "macerate" and wriggle, until all the iniquity is soaked and washed out of them. If the amount of foul *débris* is likely to swell the river above its banks, make a crevasse in the direction of Salt river, and let it debouch thence into the "bottomless pit" of the Mammoth Cave.

